# Consumption of Red Versus White Wine and Cancer Risk: A Meta-Analysis of Observational Studies

**DOI:** 10.3390/nu17030534

**Published:** 2025-01-31

**Authors:** Rachel K. Lim, Jongeun Rhee, Megan Hoang, Abrar A. Qureshi, Eunyoung Cho

**Affiliations:** 1 Department of Dermatology, The Warren Alpert Medical School, Brown University, 339 Eddy St, Providence, RI 02903, USA; rachel_lim@brown.edu (R.K.L.); jor537@mail.harvard.edu (J.R.); megan_hoang@brown.edu (M.H.); abrar_qureshi@brown.edu (A.A.Q.); 2 Department of Epidemiology, Brown School of Public Health, Providence, RI 02903, USA; 3 Channing Division of Network Medicine, Department of Medicine, Brigham and Women’s Hospital, Harvard Medical School, Boston, MA 02115, USA

**Keywords:** wine, alcohol, cancer, nutrition, meta-analysis

## Abstract

Background/Objectives: While alcoholic beverage consumption increases cancer risk, red wine has been touted as a healthier option. To address this unexplored question, we conducted a meta-analysis to summarize evidence from observational studies. Methods: A literature search of PubMed and EMBASE through December 2023 identified studies examining wine and cancer risk. A random-effects meta-analysis was performed to estimate relative risks (RRs) and 95% confidence intervals (CIs) for an association between wine intake and overall cancer risk. Results: A total of 20 cohort and 22 case–control studies were included. Wine intake was not associated with overall cancer risk (*n* = 95,923) when comparing the highest vs. lowest levels of consumption, with no differences observed by wine type (red: summary RR = 0.98 [95% CI = 0.87, 1.10], white: 1.00 [0.91, 1.10]; P_difference_ = 0.74). However, white wine intake was significantly associated with an increased risk of cancer among women (white: 1.26 [1.05, 1.52], red: 0.91 [95% CI: 0.72, 1.16], P_difference_ = 0.03) and in analyses restricted to cohort studies (white: 1.12 [1.03, 1.22], red: 1.02 [95% CI: 0.96, 1.09], P_difference_ = 0.02). For individual cancer sites, there was a significant difference in associations between red and white wine intake only in skin cancer risk [6 studies, white: 1.22 (1.14, 1.30), red: 1.02 (0.95, 1.09); P_difference_ = 0.0003]. Conclusions: We found no differences in the association between red or white wine consumption and overall cancer risk, challenging the common belief that red wine is healthier than white wine. Our significant results related to white wine intake in subgroup analyses warrant further investigation.

## 1. Introduction

Alcoholic beverages have been classified as Group 1 carcinogens (i.e., carcinogenic to humans) by the International Agency for Research on Cancer (IARC) based on sufficient evidence in humans for cancers of the oral cavity, pharynx, larynx, esophagus, liver, colorectum, and female breast [1]. According to the IARC Global Cancer Observatory database, 741,300 cancer cases in 2020, which is 4.1% of total global cancer cases for that year, were attributed to alcohol consumption. Ethanol in alcohol metabolizes into acetaldehyde, which readily forms Schiff-base adducts with DNA and cellular proteins [2], resulting in point mutations and harmful DNA–protein and DNA–DNA crosslinks [3]. It has also been found that some alcoholic beverages contain significant levels of preexisting acetaldehyde [4,5]. Despite these findings, the consumption of wine in particular has been rising [6].

It is not clear whether cancer risk differs by consumption of different types of wine, in particular red versus white. Red wine has been considered a healthier option because it contains a higher number of antioxidants, including flavonoids and polymeric tannins, than white wine [7]. In fact, a recent Canadian survey showed that 41% of respondents felt uncertain about whether or not red wine reduced cancer risk, while 54% agreed that alcohol consumption increased cancer risk [8]. Numerous experimental studies found that resveratrol, a polyphenol stilbenoid in red wine, inhibits the proliferation of several different types of cancer cells [9,10,11,12,13,14,15]. Epidemiologic studies have primarily examined the relationship between overall alcohol intake and cancer risk [16,17]; however, studies that have separately analyzed red and white wine have reported inconsistent findings. Several studies have observed inverse or no associations between red wine intake and cancers of the breast, skin, prostate, ovaries, colon, and lungs [18,19,20,21,22,23,24,25,26,27,28,29,30,31,32,33,34,35,36,37,38,39,40,41,42,43], while other studies have shown increased risk of aforementioned cancers [19,37,39,44,45,46,47]. Similarly, white wine, which generally contains a lower amount of resveratrol than red wine (0–1.09 mg/L vs. red wine: 0.36–1.97 mg/L [48]), has been positively [29,32,33,35,36,41,44], inversely, or not [34,42] associated with cancers of the breast, skin, and prostate. A systematic review and meta-analysis summarized the relationship between wine consumption and cancer risk but did not evaluate red and white wine separately [49]. To our knowledge, there are few studies evaluating the difference in cancer risk by type of wine.

To address this research gap, especially given the vast and often contradictory literature on the carcinogenicity of red and white wine separately, we conducted a meta-analysis to investigate the association between red versus white wine consumption and the risk of all and site-specific cancers. We aimed to clarify the potentially differing carcinogenic effects of red and white wine and contribute to a more nuanced understanding of the role of wine type in cancer risk. Ultimately, this research may provide valuable insights to inform public health guidelines and individual lifestyle decisions regarding wine.

## 2. Materials and Methods

### 2.1. Search Strategy

A comprehensive search of PubMed and Embase was conducted for studies published up to December 2023 using the Medical Subject Headings (MeSH) terms or keywords including cancer, tumor, carcinoma, malignant neoplasm, alcohol, wine, red wine, and white wine (Supplementary Table S1). Three independent reviewers (RL, JR, and MH) screened the titles, abstracts, and full texts based on pre-defined eligibility criteria. Any disagreements regarding study inclusion were discussed and resolved by the three investigators (RL, JR, and MH). Additionally, the reference lists of identified articles and relevant meta-analyses or reviews were examined to locate additional relevant studies. The Meta-analysis of Observational Studies in Epidemiology (MOOSE) guidelines were adhered to in the design, execution, analysis, and reporting of this meta-analysis [50].

### 2.2. Study Selection and Data Extraction

**Figure 1:**
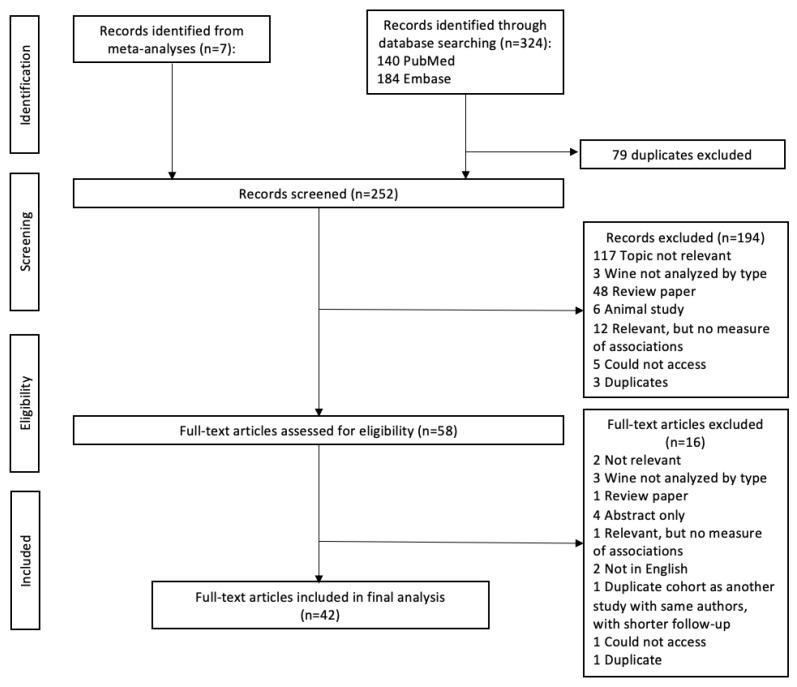
Literature search results for publications related to wine consumption (red or white) and cancer risk.

We included prospective cohort and case–control studies that examined the association between red or white wine and cancer risk. We restricted our analysis to studies providing the type of wine (red or white, rather than wine as a whole) and relevant measures of association, relative risk (RR) or odds ratio (OR) estimates with corresponding 95% confidence intervals (CIs), to perform a meta-analysis. Articles not published in English, literature reviews, abstracts, posters, case reports, and experimental studies were excluded (Figure 1). Studies with more than three categories of wine consumption, along with data on the case and non-case numbers, RR or OR estimates, and 95% CIs for each category, were eligible for inclusion in the dose–response analysis.

From each included study, we extracted the following details: author names, publication year, study design (cohort or case–control), type of wine (red or white), number of cases and non-cases, sample size, the most fully adjusted estimates of association and corresponding 95% CIs, study region, measurement of wine consumption (validated or not), measurement of outcomes (confirmed by medical record or not), and any adjustments for potential confounders (Table 1). For the dose–response meta-analysis, we collected category-specific doses of wine consumption (range, median) and the most adjusted RRs or ORs and their corresponding 95% CIs. The lowest wine intake category (nondrinkers) was used as the reference group. For each study, we assigned the midpoint of each wine intake category (range) as the corresponding RR or OR for that category. If the highest category was unbounded, we estimated the same range as the other categories for the highest category (e.g., if categories are <1, 1–3, or >3, we assumed 3–5 for the last category and assigned 4 as the midpoint). Wine intake measurements were all converted to grams of ethanol per day for dose–response analysis. The standard drink definition established by the government of the study’s country of origin was utilized to calculate conversions [51]. All extracted data were independently reviewed and cross-verified by the authors at least twice.

### 2.3. Statistical Analysis and Quality Assessment

A meta-analysis for highest versus lowest exposure levels was conducted. The summary RRs and 95% CIs were calculated by using a random-effects model. We performed subgroup analyses by type of wine (red, white) and cancer type for those having more than three studies (skin, colon/rectum, kidney/urinary tract, lung, ovary, female breast, and prostate). Then, meta-regression was used to assess whether the risks of cancer overall or site-specific cancers differ by type of wine (*p*-value for difference). We also conducted analyses restricted to cohort studies, which are less susceptible to recall or selection bias than case–control studies. A random-effects exposure–response meta-analysis was performed to examine a linear relationship between wine intake and cancer risk.

Potential publication bias was assessed through visual inspection of funnel plot asymmetry and by the *p*-value from Egger’s tests [64]. Sensitivity analyses were conducted to evaluate the cancer risk, excluding studies that only had one type of wine intake (red or white) [18,43,46,47,61]. Additionally, the analysis was repeated after removing the most influential study with results that significantly deviated from the summary estimates. Between-study heterogeneity was evaluated using Cochran’s Q statistic and quantified with Higgins I^2^ statistic and associated 95% CI [65]. Sources of heterogeneity were investigated through meta-regression and subgroup analyses, considering variables such as study design (case–control or cohort), geographic region (US/Canada or other regions), sex, publication year (1990s, 2000s), exposure measurement assessment (validated or not), and the level of confounder adjustment [smoking and body mass index (BMI)] (Table 1).

## 3. Results

The searching scheme resulted in a total of 252 published articles, and we selected 42 articles (20 cohort [20,21,22,25,26,29,31,32,35,36,37,38,41,43,44,45,53,56,60,63] and 22 case–control studies [18,19,23,24,27,28,30,33,34,39,40,42,46,47,52,54,55,57,58,59,61,62]) to conduct a meta-analysis (Figure 1, Table 1). Of the 42 studies selected, 25 used validated methods of dietary assessment [18,20,21,22,25,26,27,28,29,30,32,34,35,36,37,38,40,41,43,44,45,53,59,60,63] (Table 1). A total of thirty-seven studies included measures of association for site-specific cancer incidence and consumption of both red and white wine, and five studies included data for red wine intake only [18,43,46,47,61]. We included cancers of the skin (number of studies = 6) [29,32,36,41,42,45], prostate (*n* = 6) [20,34,35,37,38,53], female breast (*n* = 5) [19,25,30,39,44], colon/rectum (*n* = 5) [24,28,31,43,59], ovary (*n* = 5) [23,26,27,52], lung (*n* = 4) [21,22,33,46], kidney/urinary track (*n* = 3) [18,56,57], pancreas (*n* = 2) [54,55], brain (*n* = 2) [62,63], lymphoma (*n* = 2) [60,61], stomach (*n* = 1) [58], and mouth/pharynx (*n* = 1) [46]. When a study presented effect estimates for multiple cancers (different sites or a subtype) or sex-specific estimates, we included all estimates and considered them as separate studies in our analyses.

The summary RR for the overall cancer risk, comparing the highest versus lowest level of wine intake, was 0.98 (95% CI = 0.87 to 1.10) for red wine and 1.00 (95% CI = 0.91 to 1.10) for white wine (Table 2). We observed no difference between red and white wine consumption on overall cancer risk (P_difference_ = 0.74). After excluding five studies [18,43,46,47,61] that only included measurements of association for red wine, we still observed no difference in overall cancer risk between the two types of wine (P_difference_ = 0.38). When restricted to cohort studies, the association with white wine intake became stronger and significant (RR: 1.12, 95% CI: 1.03, 1.22), while the null association remained for red wine intake (RR: 1.02, 95% CI: 0.96, 1.09), and the difference became significant (P_difference_ = 0.02).

The difference between red and white wine consumption on overall cancer risk was not significant in men (P_difference_ = 0.37) but was significant in women (P_difference_ = 0.03). The eligible studies for linear dose–response analyses were three cohort studies [22,25,38] and twelve case–control studies [18,19,23,25,30,34,39,47,52,54,57,58,59]. Based on these studies, we observed that every additional 10 g of estimated ethanol (e.g., about one glass) from red wine per day was associated with a 5% increase in overall cancer risk (summary RR = 1.05 [1.03, 1.08]); however, this association was null when restricted to cohort studies (1.01 [0.97, 1.04]) [21,24,37]. We found no significant dose–response relationship for white wine (RR = 1.02 [0.98, 1.05]), which was consistent when restricting to cohort studies (RR = 1.00 [0.96, 1.04]).

**Figure 2:**
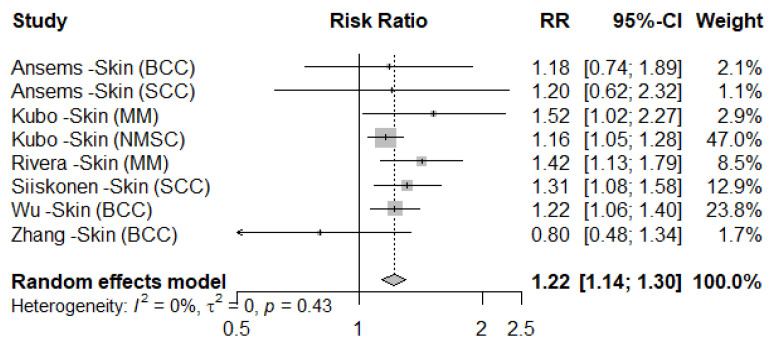
Forest plot of meta-analysis using random effects model for white wine consumption associated with skin cancer risk (comparing highest vs. lowest intake) [29,32,36,41,42,45]. The rectangles represent 95% confidence intervals and the diamond signifies the pooled effect of all included studies. Abbreviations: BCC, basal cell carcinoma; SCC, squamous cell carcinoma; MM, malignant melanoma.

When we evaluated the association between wine intake and cancer risk by cancer site, we observed a significant difference in skin cancer risk associated with red versus white wine (P_difference_ = 0.0003). White wine intake was associated with a 22% increased risk of skin cancer (RR = 1.22, 95% CI = 1.14 to 1.30, Table 2, Figure 2), while red wine consumption was not associated with skin cancer (RR = 1.02, 95% CI = 0.95 to 1.09). Studies of skin cancer were not eligible to assess the dose–response relationship. An increased risk of breast cancer was observed for both wine types (red: RR = 1.17, 95% CI = 0.97 to 1.42, white: 1.12, 95% CI = 1.05 to 1.20) with no significant difference (P_difference_ = 0.61). Other than skin cancer, we did not observe a significant difference between the associations of red and white wine consumption and the risk of site-specific cancers.

The test of heterogeneity resulted in a moderate level of heterogeneity (I^2^ = 63.9%, P_heterogeneity_ < 0.0001) (Table 3). After excluding the most influential study [39], there was no change in directionality and significance of the summary RRs of high vs. low meta-analysis for overall cancer risk (red: 0.96, 95% CI: 0.86, 1.08; white: 1.00, 95% CI: 0.91, 1.10) and no significant difference between red and white wine (*p* = 0.63).

We observed possible publication bias through visual inspection of the funnel plot (Supplementary Figure S1) and Egger’s tests (*p* = 0.03). We used Duval and Tweedie’s trim and fill method [66] to estimate what the summary effect size would be if there was no publication bias. Briefly, this method omits small studies until the funnel plot is symmetrical (“trimming”), uses the trimmed plot to estimate the “true” center of the funnel plot, and then puts back the omitted studies, as well as their theoretical missing “counterparts”, around the new center (“filling”). We observed slightly increased summary RRs for both red and white wine on overall cancer risk after conducting trim and fill analysis (red: 1.00, 95% CI 0.91, 1.09; white: 1.13, 95% CI 1.03, 1.24) (Supplementary Figure S2).

## 4. Discussion

We conducted a meta-analysis to investigate the association between red or white wine consumption and cancer risk. Although red wine has been considered healthier compared to white wine [67,68,69,70], there was no difference in cancer risk between the consumption of the two types of wine. When we restricted analysis to cohort studies, we observed a significant increase in overall cancer risk with white wine consumption. White wine intake was significantly associated with an increased risk of skin cancer compared to red wine intake.

A recent UK cohort study evaluating alcohol consumption patterns and health outcomes found no significant increase in the overall cancer risk and alcohol-related cancer (colon, rectum, breast, liver, esophagus, and larynx) incidence with white wine consumption compared to that of red wine, which is in line with our findings [71]. Red wine contains resveratrol, a natural stilbene and a non-flavonoid polyphenol, that possesses antioxidant, anti-inflammatory, cardioprotective, and anti-cancer properties [72]. Resveratrol has been investigated extensively as a potential chemopreventive agent because it has been shown to inhibit the proliferation of cancer cells in the breast, colon/rectum, skin, stomach, and kidney [9,10,11,12,13,14]. Many in vitro studies found that resveratrol influences tumor initiation and cancer progression pathways; however, in vivo studies have yielded mixed results [73]. For example, resveratrol can promote cell-cycle arrest leading to apoptosis of tumor cells, prevent tumor-derived nitric oxide synthase expression to block tumor growth and migration, as well as act as an antioxidant to prevent DNA damage that can lead to tumor formation [73]. Wu et al. [74] found that resveratrol is more toxic to cancer cell lines than to normal cell lines by comparing the activity of both types of cells following treatment with resveratrol. Additionally, it has been observed that combination therapy of resveratrol and other cancer drugs kills breast cancer cells more dramatically than either treatment in isolation [75]. Although there is some evidence of an anticarcinogenic effect of resveratrol in experimental studies, we did not observe that red wine consumption is associated with a reduced risk of cancer in our meta-analysis. In addition, there was no difference in the association between red vs. white wine and cancer risk, except for skin cancer.

A potential reason why we did not observe a difference in cancer risk between red and white wine may be that resveratrol has a fast metabolism [76]. Although concentrations of resveratrol are greater in red compared to white wine [48,77], studies have demonstrated that resveratrol is metabolized quickly, with nearly 75% excreted via feces and urine [76]. Several clinical studies have shown that the peak levels of resveratrol in plasma were low, given its extensive metabolism and poor bioavailability, after single or repeated oral administration [78]. A study involving 15 healthy participants who were given a single dose of 500 mg resveratrol observed a peak plasma concentration of 71.2 ng/mL [79]. Another study of 40 healthy subjects who received daily oral resveratrol of 500–5000 mg/day for 29 days reported a peak plasma concentration ranging from 44 to 967 ng/mL between days 21 and 28 [80]. Daily consumption of two glasses of red wine (375 mL) is equivalent to a dose of ~27 µg/kg body weight of resveratrol for a 70 kg individual, which leads to detectable concentrations of derivatives but not free resveratrol [81]. These low levels of resveratrol after consuming red wine may not result in bio-effective concentrations that may eventually make differences in health outcomes compared to white wine consumption.

We observed that white wine was associated with an increased risk of skin cancer. It has been hypothesized that alcohol consumption can promote skin carcinogenicity through the intermediate byproducts or metabolites of alcohol, such as acetaldehyde and reactive oxygen species, that have photosensitizing effects [82]. Also, drinking wine may be more common among whites who are more susceptible to skin cancer than other racial groups [68,83]. However, it may not explain why white wine specifically was associated with skin cancer risk. In addition, heavy wine consumption may be related to high-risk behaviors such as sunburn, indoor tanning, and lack of use of sun protection [84]. In fact, earlier studies have demonstrated an association between alcohol consumption and increased prevalence of severe sunburn [82,85]. Given that all six studies properly adjusted for detailed information on the risk factors of skin cancer, e.g., skin type, sun exposure, and sun protection habits, a positive association we found in the meta-analysis was most likely the true detrimental effect of white wine [29,32,36,41,42,45]. The sample size for skin cancer was the largest among individual cancer sites and potentially provided enough statistical power to detect the difference. Wine intake (both red and white) was associated with increased breast cancer risk, which may suggest that resveratrol is not a significant factor in breast carcinogenesis.

There are limitations to this meta-analysis. First, wine intake was obtained from a self-reported food frequency questionnaire (FFQ). While most of the included cohort studies used validated FFQs (18 out of 19), only six out of twenty-two case–control studies used validated FFQs. However, because the intake information on red and white wine was collected in each study, the misclassification would similarly affect the intake assessment of both wines. Nevertheless, the misclassification of exposure might have occurred randomly regardless of cancer status, which may attenuate our findings. In addition, exposure validation was not a source of heterogeneity in our summary estimates for both red and white wine. Additionally, case–control studies may be more susceptible than cohort studies to recall bias when evaluating wine intake; however, we also presented results restricted to cohort studies. For some cancer sites including the pancreas, lymphatic system, brain, mouth, and pharynx, there was a limited number of studies (<3) to explore the risk difference between red and white wine. In addition, our dose–response analyses were limited to a smaller number of studies, largely case–control studies. Potential regression dilution bias in the included cohort studies might have also played a role in underestimating summary risk estimates [86].

However, we added an important finding to the controversial conversation about whether red wine intake reduces cancer risk. The quality of outcome measurements in all included studies was reliable (from a cancer registry or confirmed by medical professionals). Although we found evidence of potential publication bias, we found similar summary estimates after conducting trim and fill analysis.

## 5. Conclusions

To our knowledge, we conducted the first meta-analysis and the largest investigation of red versus white wine consumption and cancer risk and found no difference between the two types of wine. However, we observed that white wine had a significantly stronger association with cancer when the analysis was restricted to cohort studies. Furthermore, white wine intake, but not red wine intake, was associated with an increased risk of skin cancer. Our findings provided a critical public health message that drinking red wine may not be any better than drinking white wine in terms of cancer risk.

## Figures and Tables

**Table 1:** Summary table of 42 selected studies in the meta-analysis.

Cancer Type	Author, Year	Country	Study Design	Population	Measurement of Wine Intake (Used Validated Food Frequency Questionnaire)	Number of Cases/Number of Participants	Adjusted Confounders
Skin (BCC, SCC)	Ansems, 2008 [45]	Australia	Cohort (Nambour Skin Cancer Study)	Adult residents of Nambour, Queensland, Australia, average age of 50 years	Yes	394/1360	BCC: age, sex, beta-carotene treatment, sunscreen treatment, elastosis of the neck, occupational sun exposure, leisure time sun exposure, history of skin cancer before 1992
							SCC: age, sex, beta-carotene treatment, sunscreen treatment, self-reported skin color, number of pack-years smoked until 1992, elastosis of the neck, leisure time sun exposure, history of skin cancer before 1992
Skin (MM, NMSC)	Kubo, 2014 [29]	USA	Cohort (Women’s Health Initiative)	White postmenopausal women aged 50–79 from across the USA	Yes	10,125/59,575	Age, education, BMI, Langleys of exposure, physical activity, history of NMSC, history of MM, smoking, sunscreen use, current summer sun exposure, childhood sun exposure, skin reaction to the sun, last medical visit within 1 year, having a current care provider, having insurance
Skin (BCC)	Zhang, 2014 [42]	USA	Case–control	Individuals aged <40 in Connecticut diagnosed with BCC and matched controls	No	365/747	Age, gender, body site of biopsy, indoor tanning, skin color, education, smoking status, hours spent outdoors in warm months, sunburns, family history of skin cancer, consumption of other types of alcohol
Skin (BCC)	Wu, 2015 [41]	USA	Cohort (Nurses’ Health Study, Nurses’ Health Study II, Health Professionals Follow-Up Study)	Female registered nurses aged 25–55, male health professionals	Yes	15,681/211,462	Age, BMI, smoking status, physical activity, caffeine intake, ethnicity, family history of melanoma, natural hair color, number of moles on arms or legs, skin reaction to sun as a child/adolescent, number of severe sunburns, cumulative UV flux since baseline, Average time spent in direct sunlight in summer months, use of sunscreen in summer months, consumption of other types of alcohol
Skin (SCC)	Siiskonen, 2016 [36]	USA	Cohort (Nurses’ Health Study, Nurses’ Health Study II, Health Professionals Follow-Up Study)	Female registered nurses aged 25–55, male health professionals	Yes	2938/223,138	Age, BMI, smoking status and pack-years smoked, physical activity, caffeine intake, family history of melanoma, tanning ability, lifetime number of severe sunburns, number of moles, natural hair color, average annual UV-B flux at place of residence, total alcohol intake
Skin (MM)	Rivera, 2016 [32]	USA	Cohort (Nurses’ Health Study, Nurses’ Health Study II, Health Professionals Follow-Up Study)	Female registered nurses aged 25–55, male health professionals	Yes	2209/21,052	Age, BMI, smoking status, physical activity, caffeine intake, family history of melanoma, tanning ability, lifetime number of severe sunburns, number of moles on forearms, hair color at age 18, average annual UV-B flux at place of residence, intake of other alcoholic beverages
Ovary	Webb, 2004 [40]	Australia	Case–control	Women aged 18–79 with epithelial ovarian cancer in Australia	Yes	696/1482	Age, age squared, level of education, BMI, smoking status, duration of oral contraceptive use, parity, caffeine intake, consumption of other types of alcohol
Ovary	Kelemen, 2004 [26]	USA	Cohort (Iowa Women’s Health Study)	Women aged 55–69 randomly selected from Iowa driver’s license registry	Yes	147/27,205	Age at menopause, physical activity, postmenopausal hormone use, oral contraceptive use, family history of breast cancer, family history of ovarian cancer, known diabetes at baseline, smoking, energy-adjusted intakes of total carotene, vitamin C and vitamin E
Ovary	Kelemen, 2013 [27]	USA	Case–control	Pooled data from 12 case–control studies performed in USA, Canada, Europe, and Australia; all of used population-based ascertainment methods for identifying cases and control	Yes	5342/10,358	Age, smoking status, site, race/ethnicity, menopausal status, oral contraceptive use, tubal ligation, endometriosis, hysterectomy, Family history of breast or ovarian cancer in first-degree relatives, parity/age at last birth, interview year, age at menarche, BMI, education
Ovary	Cook, 2016 [23]	Canada	Case–control	Women aged 20–79 diagnosed with ovarian tumors and matched controls	No	1144/3657	Age, oral contraceptive use, parity, current smoking, family history of ovarian or breast cancer
Ovary	L’Espérance, 2023 [52]	Canada	Case–control	Women aged 18–79 years of the Greater Montreal area with ovarian cancer	No	495/1397	Age, number of full-term pregnancies, education, other type of alcoholic beverage intake
Prostate	Schuurma, 1999 [35]	Netherlands	Cohort (Netherlands Cohort Study)	Men aged 55–69	Yes	680/58,279	Age, family history of prostate cancer, socioeconomic status, total alcohol intake
Prostate	Schoonen, 2005 [34]	USA	Case–control	European and African American men aged 40–64 in King County, WA	Yes	753/1456	Age, PSA screening, total lifetime number of female sexual partners, smoking status, consumption of other types of alcohol
Prostate	Velicer, 2006 [38]	USA	Cohort (Vitamins and Lifestyle (VITAL) Cohort)	Men aged 50–76 in Washington State	Yes	816/34,565	Age, PSA test within 2 years of baseline, consumption of other types of alcohol
Prostate	Sutcliffe, 2007 [37]	USA	Cohort (Health Professionals Follow-Up Study)	Male health professionals aged 40–75	Yes	3348/45,433	Age, race/ethnicity, BMI at 21, cumulative family history of prostate cancer through 1996, height, updated cigarette smoking in the past 10 years, baseline intakes of total energy, tomato sauce, red meat, fish, calcium and vitamin E, baseline energy-adjusted intakes of fructose and α-linolenic acid, baseline vigorous physical activity, diabetes mellitus type 2, vasectomy
Prostate	Chao, 2010 [20]	USA	Cohort (California Men’s Health Study)	Men aged 45–69 who were members of Kaiser Permanente health plans in California	Yes	1340/65,972	Red wine: age, race/ethnicity, income, BMI, intake of other alcoholic beverages, meat consumption, family history of prostate cancer, person history of PSA testing, STI, BPH, BPH surgery, prostatitis, diabetes mellitus
							White wine: race, income, BMI, intake of other alcoholic beverage, meat consumption, family history of prostate cancer, personal history of PSA testing, STI, BPH, BPH surgery, prostatitis, diabetes
Prostate	Downer, 2019 [53]	USA	Cohort (Health Professionals Follow-Up Study)	Male health professionals aged 40–75 who were diagnosed with nonmetastatic prostate cancer during follow-up	Yes	5182/47,568	Total energy intake, smoking, BMI, vigorous physical activity, choline, vegetable fat, coffee, lycopene, whole milk, and diabetes, prostate-specific antigen (PSA) screening beginning in 1994, diabetes. Beer, wine, and liquor were mutually adjusted for each other.
Pancreatic	Farrow, 1990 [54]	USA	Case–control	Men aged 20–74 diagnosed with pancreatic cancer and matched controls	No	148/336	Age, smoking, race, education
Pancreatic	Bueno de Mesquita, 1992 [55]	Netherlands	Case–control	Individuals aged 35–79 living in the central Netherlands diagnosed with cancer of the exocrine pancreas and matched controls	No	176/663	Age, sex, response status, lifetime smoking of cigarettes, dietary intake of energy and vegetables, lifetime consumption of other alcoholic drinks
Urinary Tract	Andreatta, 2010 [18]	Argentina	Case–control	Patients with urinary tract tumors in Cordoba, Argentina, and controls taken from hospital registries at the time of diagnosis of cases	Yes	168/502	Age, sex, BMI, tobacco smoking, occupational exposure, social status, physical activity
Renal	Nicodemus, 2003 [56]	USA	Cohort (Iowa Women’s Health Study)	Women aged 55–69 randomly selected from Iowa driver’s license registry	Yes	124/34,637	Age
Renal	Greving, 2007 [57]	Sweden	Case–control	Men and women aged 20–79 diagnosed with renal cancer and born and living in Sweden; controls were randomly selected and matched to cases	No	855/2059	Age, sex, BMI, cigarette smoking, consumption of other types of alcohol
Breast	Viel, 1997 [39]	France	Case–control	Women aged 30–50 diagnosed with breast cancer without previous specific treatment	No	154/308	Total calorie intake, parity
Breast	Hirvonen, 2006 [25]	France	Cohort (French Supplementation en Vitamines et Mine raux Antioxydants Study)	Women aged 35–60 and men 45–60 without a history of cancer	Yes	95/4396	Age, smoking, number of children, use of oral contraception, family history of breast cancer, menopausal status
Breast	Allen, 2009 [44]	United Kingdom	Cohort (Million Women Study)	Women who attended breast cancer screenings in the UK	Yes	28,380/1,280,296	Age, region of residence, socioeconomic status, BMI, smoking status, physical activity, use of oral contraceptives and hormone replacement therapy
Breast	Newcomb, 2009 [30]	USA	Case–control	Women aged 20–69 diagnosed with breast cancer and matched controls	No	6327/13,885	Age, state, family history of breast cancer, age at menarche, age at first birth, parity, menopausal status, age at menopause, postmenopausal hormone use, BMI, education, total alcohol consumption
Breast (ductal, ductal-lobular, lobular)	Baglia, 2017 [19]	USA	Case–control	Women aged 55–74 diagnosed with breast cancer in the Greater Seattle area and matched controls	No	1961/2852	Age, reference year, county of reference
Mouth/Pharynx	De Stefani, 2007 [47]	Uruguay	Case–control	Patients and controls recruited from hospitals in Montevideo, Uruguay	No	776/2277	Age, residence, urban/rural status, hospital, year at diagnosis, education, family history of cancer among first-degree relatives, occupation, total vegetables and fruits consumption, maté intake, smoking status, years since quit, cigarettes/day among current smokers, total alcohol intake
Lung	De Stefani, 1993 [46]	Uruguay	Case–control	Patients and controls recruited from hospitals in Montevideo, Uruguay	No	327/677	Age, residence, education, cigarette smoking measured in pack-years
Lung	Ruano-Ravina, 2004 [33]	Spain	Case–control	Lung cancer patients in Santiago University Teaching Hospital and matched controls	No	132/319	Age, sex, occupation, smoking habit, total alcohol intake
Lung	Chao, 2008 [22]	USA	Cohort (California Men’s Health Study)	Men aged 45–69 who were members of Kaiser Permanente health plans in California	Yes	210/84,170	Age, ethnicity, household income, BMI, smoking status, cigarettes smoked per day, smoking duration, and history of COPD/emphysema
Lung	Chao, 2011 [21]	USA	Cohort [Vitamins and Lifestyle (VITAL) Study]	Men and women aged 50–76 living in western Washington State covered by SEER registry	Yes	580/66,186	Gender, race, education, household income, BMI, history of COPD/emphysema, cigarette smoking, family history of lung cancer, high-intensity physical activity, fat intake, fruit and vegetable intake
Stomach	Falcao, 1994 [58]	Portugal	Case–control	Patients undergoing gastroscopy	No	74/267	Social class, milk consumption, number of meals/day, rice consumption, vegetable consumption
Colon/ rectum	Chao, 2010 [43]	USA	Cohort (California Men’s Health Study)	Men aged 45–69 who were members of Kaiser Permanente health plans in California	Yes	176/43,483	Crude
Colon/ rectum	Kontou, 2012 [28]	Greece	Case–control	Men and women diagnosed with CRC at a hospital in Athens, Greece	Yes	250/500	Age, BMI, current smoking, physical activity, family history of colorectal cancer
Colon/ rectum	Grosso, 2014 [24]	Italy	Case–control	Men and women diagnosed with CRC recruited from the list of patients who visited a hospital in Catania, Southern Italy	No	338/1014	Age, sex, smoking status, family history of CRC, obesity status, having diabetes, physical activity level, alcohol quantity
Rectum	Murtaugh, 2004 [59]	USA	Case–control	Men and women from the Northern California Kaiser Permanente aged 30–79	Yes	952/2157	Energy, fiber and calcium intake, age, physical activity
Colon	Phipps, 2016 [31]	USA	Cohort (Multicenter Phase III drug trial led by North Central Cancer Treatment Group)	Patients with resected stage III colon cancer	No	649/1984 (outcome is colon cancer recurrence or death)	Treatment, sex, BMI, smoking, physical activity, performance score, race
Lymphoma (non-Hodgkin lymphoma)	Chiu, 1999 [60]	USA	Cohort (Iowa Women’s Health Study)	Women aged 55–69 randomly selected from Iowa driver’s license registry	Yes	143/35,156	Age, residence, education, marital status, transfusion history, diabetes history, intake of red meat, intake of fruits, total energy intake
Lymphoma	De Stefani, 2013 [61]	Uruguay	Case–control	Men and women diagnosed with lymphoid cancers in Montevideo, Uruguay, with matched controls	No	697/4303	Age, sex, residence, urban/rural status, education, BMI, smoking intensity in pack-years, total meat, total vegetable, and fruit intake, total meat, total energy
Brain (glioma, meningioma)	Ryan, 1992 [62]	Australia	Case–control	Adults aged 25–74 diagnosed with primary tumors of the brain and meninges in metropolitan Adelaide, Australia	No	170/587	Age, sex
Brain (glioma, glioblastoma)	Cote, 2021 [63]	USA	Cohort (Nurses’ Health Study, Nurses’ Health Study II, Health Professionals Follow-Up Study)	Female registered nurses aged 25–55, male health professionals	Yes	554/237,505	Age, calendar year, smoking status, BMI, total caloric intake

Abbreviations: BCC, basal cell carcinoma; BMI, body mass index; BPH, benign prostatic hyperplasia; COPD, chronic obstructive pulmonary disease; CRC, colorectal cancer; MM, malignant melanoma; NMSC, non-melanoma skin cancer; PSA, prostate-specific antigen; SCC, squamous cell carcinoma; STI, sexually transmitted infection. Murtaugh et al. [57] only presented effect estimates for women and men separately; we added both estimates and considered them as separate studies. For studies that provided estimates for all sex, women only, and/or men only, we included estimates for all sex. When a study provided results for individual cancer subtypes (instead of overall risk) or different kinds of cancers, we included all estimates and considered them as separate studies (e.g., Baglia et al. [45] (breast cancer): ductal, lobular, and ductal-lobular; Kubo et al. [28] (skin cancer): MM and NMSC; De Stefani et al. [46]: oral and pharyngeal cancer). Andreatta et al. [44], De Stefani et al. [23], De Stefani et al. [64], De Stefani et al. [46], and Chao et al. [42] only provided results of red wine intake.

**Table 2:** Summary RRs and 95% CIs of cancer risk associated with red and white wine, overall and stratified by sex and cancer sites.

	Red Wine			White Wine			P_difference_
	No. of Studies	No. of Cases /No. of Participants	Summary RR (95% CI)	No. of Studies	No. of Cases /No. of Participants	Summary RR (95% CI)	
All cancer	42	95,923/2,824,425	0.98 (0.87, 1.10)	37	95,747/2,780,942	1.00 (0.91, 1.10)	0.74
Sex							
Men	13	10,300/678,821	1.01 (0.91, 1.12)	10	8906/484,051	1.08 (0.99, 1.18)	0.37
Women	15	61,211/1,723,992	0.91 (0.72, 1.16)	15	61,211/1,723,992	1.26 (1.05, 1.52)	0.03
Cancer sites							
Female breast	5	36,917/1,301,737	1.17 (0.97, 1.42)	5	36,917/1,301,737	1.12 (1.05, 1.20)	0.61
Skin	6	31,712/706,534	1.02 (0.95, 1.09)	6	31,712/706,534	1.22 (1.14, 1.30)	0.0003
Ovary	5	7676/44,099	0.76 (0.46, 1.26)	5	7676/44,099	0.95 (0.81, 1.11)	0.76
Prostate	6	12,119/253,273	0.92 (0.71, 1.18)	6	12,119/253,273	1.08 (0.72, 1.60)	0.51
Lung	4	1249/151,352	0.82 (0.41, 1.63)	3	922/150,675	0.79 (0.52, 1.22)	0.94
Kidney/Urinary tract	3	1147/37,198	0.79 (0.44, 1.42)	2	979/36,696	0.67 (0.49, 0.92)	0.65
Colon/rectum	5	2365/49,138	0.91 (0.62, 1.32)	4	2189/5655	1.01 (0.78, 1.33)	0.64

Abbreviations: CI, confidence interval; RR, relative risk. We included each effect estimate for different subtypes of breast cancer (ductal, ductal-lobular, and lobular) in the study by Baglia et al. [45] We assumed wine intake in De Stefani et al. [46] as red wine intake since the authors mentioned that Uruguayans drink mainly red wine.

**Table 3:** Subgroup meta-analyses of wine intake (all types) and overall cancer risk comparing the highest vs. lowest levels.

Subgroups	No. of Studies	No. of Cancer Cases /No. of Participants	Summary RR (95% CI)	*I* ^2^	P_difference_
All cancer	42	95,923/2,824,425	0.99 (0.91, 1.06)	61.3%	
Study design					0.10
Cohort	20	73,771/2,772,622	1.06 (1.99, 1.13)	50.5%	
Case–control	22	22,152/51,803	0.94 (0.83, 1.07)	67.7%	
Study region					0.39
US/Canada	24	56,219/1,454,778	1.03 (0.97, 1.13)	49.2%	
Other regions	18	39,704/1,369,647	0.95 (0.80, 1.13)	74.8%	
Sex ^a^					0.93
Men	13	10,300/678,821	1.10 (0.90, 1.33)	62.2%	
Women	15	61,211/1,723,992	1.09 (1.05, 1.13)	57.5%	
Publication year					0.92
1990s	8	1872/96,273	0.98 (0.69, 1.37)	75.8%	
2000s	34	94,051/2,728,152	0.99 (0.93, 1.06)	58.4%	
Exposure validation					0.28
Yes	25	81,283 /2,787,093	1.04 (0.96, 1.12)	56.5%	
No	17	15,975/37,332	0.96 (0.85, 1.08)	67.8%	
Adjustments for confounders					0.97
Smoking and BMI	3	67,057/2,407,152	0.98 (0.86, 1.12)	71.3%	
Smoking or BMI	13	12,068/122,604	1.00 (0.84, 1.19)	64.3%	
No smoking or BMI adjustment	25	18,133/294,669	0.98 (0.88, 1.09)	58.2%	

Abbreviations: BMI, body mass index; CI, confidence interval; RR, relative risk. ^a^ Studies that provided sex-specific effect estimates are included.

## Data Availability

The data that support the findings of this study are available upon email request.

## Supplementary Materials

The following supporting information can be downloaded at: https://www.mdpi.com/article/10.3390/nu17030534/s1, Table S1: Medical Subject Heading (MeSH) terms and title/abstract (tiab) keywords in PubMed and Embase search strategy through December 2023; Figure S1: Funnel plot of meta-analysis on wine intake (highest vs. lowest) and risk of all cancer (A) All wine (*p* = 0.03) (B) Red wine (*p* = 0.04) (C) White wine (*p* = 0.04); Figure S2: Funnel plot of meta-analysis on wine intake (highest vs. lowest) and risk of all cancer after conducting trim-and-fill analysis.
