# Supplementary material for: Consumption of Red Versus White Wine and Cancer Risk: A Meta-Analysis of Observational Studies
Source: Nutrients. 2025 Jan 31;17(3):534. doi: 10.3390/nu17030534 (PMC11820282; doi:10.3390/nu17030534)
Supplement: Supplementary file 1 [file nutrients-17-00534-s001.zip › nutrients-3432188-supplementary.pdf]

Supplementary Materials

Table S1. Medical Subject Heading (MeSH) terms and title/abstract (tiab) keywords in PubMed and Embase search strategy through December 2023.

**PubMed**<br>("neoplasms"[MeSH Terms] OR cancer*[tw] OR neoplasm*[tw] OR tumor*[tw] OR carcinoma*[tw] OR malignant*[tw]) AND (red wine*[tw] OR white wine*[tw]) AND ("Epidemiologic Studies"[Mesh] OR epidemiolog*[tw] OR cohort*[tw] OR case-control*[tw] OR observational*[tw]) NOT ("Animals"[Mesh] NOT ("Animals"[Mesh] AND "Humans"[Mesh]))
**Embase**<br>('white wine':ti,ab,kw OR 'red wine':ti,ab,kw OR 'white wine'/exp OR 'red wine'/exp) AND ('malignant neoplasm':ti,ab,kw OR 'cancer':ti,ab,kw OR 'tumor':ti,ab,kw OR 'tumour':ti,ab,kw OR 'carcinoma':ti,ab,kw OR 'malignant neoplasm'/exp) AND ('epidemiology':ti,ab,kw OR 'cohort':ti,ab,kw OR 'case control':ti,ab,kw OR 'observational':ti,ab,kw OR 'epidemiology'/exp) AND 'human'/exp

(A) All wine (p=0.03)

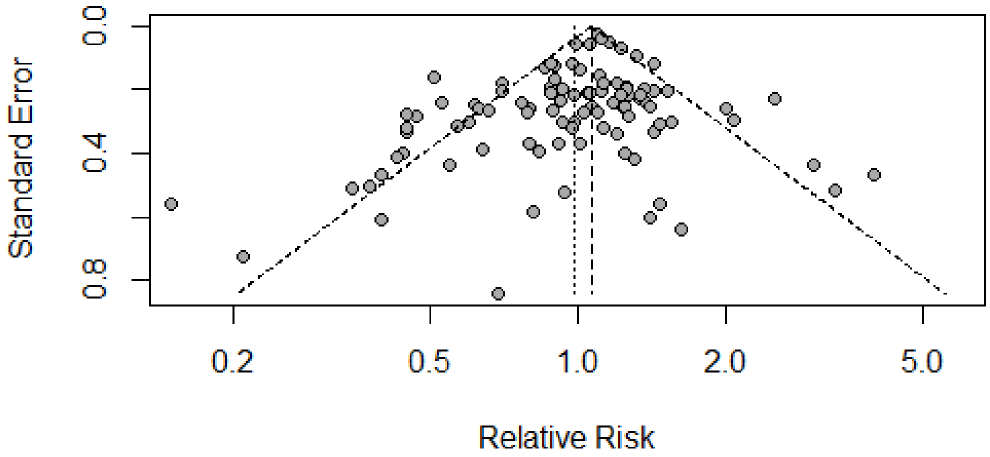

(B) Red wine (p=0.04)

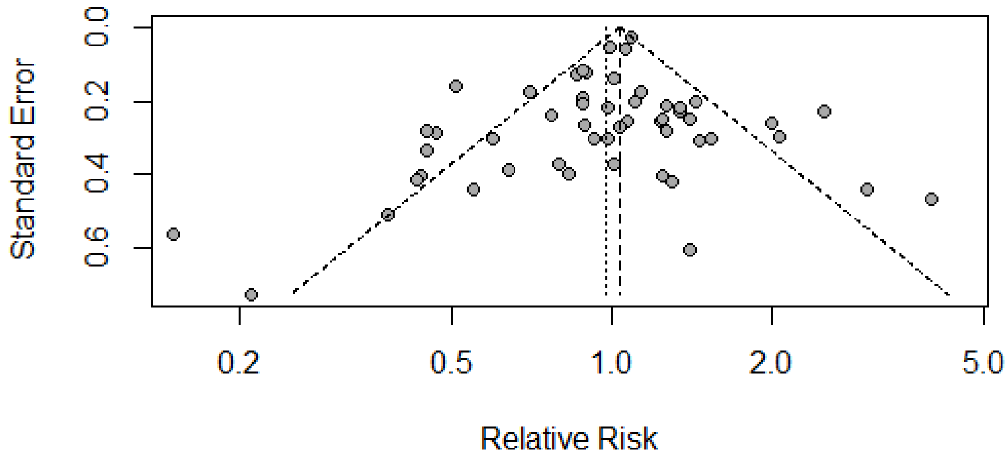

(C) White wine (p=0.04)

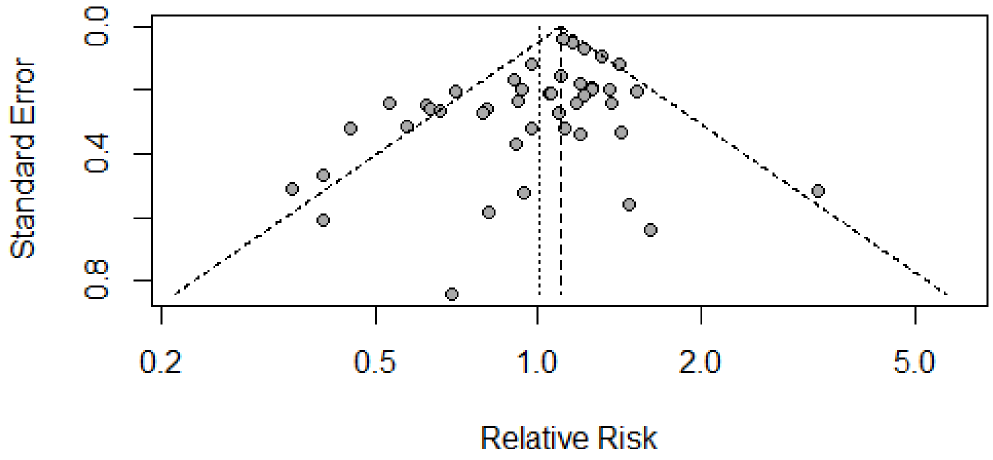

p-value is from Egger's test

Figure S1. Funnel plot of meta-analysis on wine intake (highest vs. lowest) and risk of all cancer.

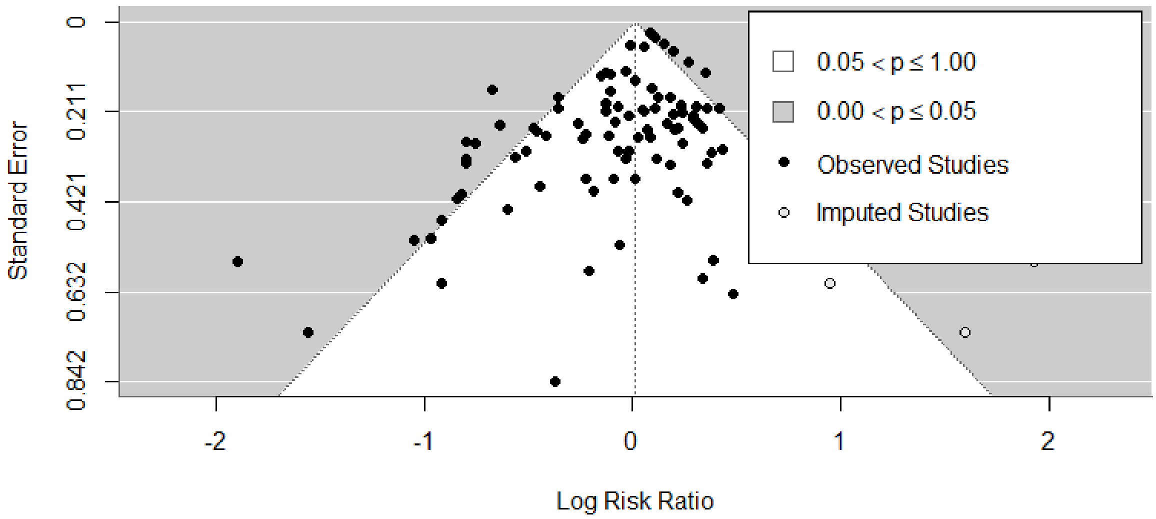

Figure S2. Funnel plot of meta-analysis on wine intake (highest vs. lowest) and risk of all cancer after conducting trim-and-fill analysis.
